# Sex-Specific Real-Life Profiling in Vedolizumab, Ustekinumab, and Tofacitinib Effectiveness in Ulcerative Colitis

**DOI:** 10.3390/jcm14217476

**Published:** 2025-10-22

**Authors:** Antonio Tursi, Raffaele Pellegrino, Giammarco Mocci, Edoardo Vincenzo Savarino, Giovanni Maconi, Walter Elisei, Antonietta Gerarda Gravina, Italian Network for Inflammatory Bowel Diseases (IN-IBD)

**Affiliations:** 1 Barletta-Andria-Trani (BAT) Local Health Agency, 76123 Andria, Italy; 2 Department of Medical and Surgical Sciences, Catholic University of Rome, 00168 Rome, Italy; 3 Hepatogastroenterology Division, Department of Precision Medicine, University of Campania Luigi Vanvitelli, 80138 Naples, Italy; raffaele.pellegrino@unicampania.it (R.P.); antoniettagerarda.gravina@unicampania.it (A.G.G.); 4 Division of Gastroenterology, AORN “Brotzu” Hospital, 09134 Cagliari, Italy; giammarcomocci@aob.it; 5 Gastroenterology Unit, Azienda Ospedale-Università di Padova (AOUP), 35128 Padua, Italy; edoardo.savarino@unipd.it; 6 Gastroenterology Unit, Department of Biomedical and Clinical Sciences, “L. Sacco” University Hospital, 20157 Milan, Italy; giovanni.maconi@unimi.it; 7 Division of Gastroenterology, A.O. “S. Camillo-Forlanini”, 00152 Rome, Italy; welisei@sancamilloforlanini.rm.it

**Keywords:** sex, ulcerative colitis, tofacitinib, vedolizumab, ustekinumab

## Abstract

**Background**: This study aimed to explore whether differences exist between males and females in a cohort of bio-experienced UC patients treated with vedolizumab (VDZ), ustekinumab (UST), or tofacitinib (TOFA) in a 48-week retrospective study. **Methods**: We evaluated intra- and inter-treatment sex-specific differences regarding clinical response, remission, steroid-free remission, sustained clinical response, late remission, and changes in faecal calprotectin and inflammatory markers at 8, 24, and 48 weeks, as well as endoscopic response and remission at 48 weeks. **Results**: Among 602 patients (50.2% female), males treated with UST had higher rates of clinical (*p* = 0.029) and steroid-free clinical remission (*p* = 0.013) at 24 weeks. Conversely, females on TOFA showed higher clinical remission at 8 weeks (*p* = 0.043). In males, VDZ demonstrated a superior clinical response over time (*p* < 0.05), while TOFA showed the highest remission rate at 48 weeks. In females, TOFA was superior for clinical remission at 8 and 24 weeks (*p* < 0.05). Males had a higher late remission rate (*p* = 0.04) with an increased likelihood (aOR 1.958, 95%CI 1.088–3.524, *p* = 0.025). Endoscopic outcomes and faecal calprotectin levels showed no significant sex-specific differences. **Conclusions**: Sex-based profiling may guide individualised therapeutic strategies in UC patients in this setting.

## 1. Introduction

Crohn’s disease (CD) and ulcerative colitis (UC) are the two main phenotypes of inflammatory bowel disease (IBD), a group of chronic, relapsing–remitting, immune-mediated diseases [1]. As in other immune-mediated diseases, sex may affect the disease onset, course, and complications, including compliance with medical and surgical therapies [2]. However, the literature concerning sex and IBD is relatively limited and, to some extent, contradictory. The male-to-female incidence ratio is estimated to be around 1:1.5 for IBD [3]. The female sex appears to be a promoting factor in CD onset, whereas it may exert a protective role in UC [4,5]. Female sex seems to be a risk factor for earlier recurrence of CD after surgery [3,6], but it seems to reduce the risk for IBD-associated colorectal cancer [7]. In patients with UC, moreover, extensive population-based studies have identified specific sex-based differences, with female sex emerging as an independent risk factor for an increased likelihood of extraintestinal manifestations and, conversely, as a protective factor against extensive disease and abdominal surgery [4]. Hormones and sex-specific differences in the immune system have been advocated as pivotal factors in the onset and subsequent development of IBD, as well as in explaining these differences [8]. Moreover, deregulation of oestrogen receptors has been observed in the intestinal context of patients with IBD, suggesting that signalling mediated by these hormones, which display well-recognised sex-related differences, may play a role in the immune system and in intestinal tissue homeostasis [9]. In addition, this has also been suggested in murine models [10]. Finally, Mendelian randomisation studies, such as the recent work by Zou et al., have also indicated that oestradiol and total testosterone may protect against CD. That oestradiol may play a protective role in males with IBD, as does total testosterone in females with IBD [11].

Nonetheless, specific UC-driving inflammatory cytotypes, which differ by sex, have also been implicated as potential variables influencing therapeutic response [12].

The sex-based profiling associated with the effectiveness of currently approved drugs for the management of IBD is significantly lacking in the literature, with minimal representation in the few available studies [13]. Indeed, there is a notable scarcity of studies specifically aimed at dissecting the differences between males and females in achieving therapeutic outcomes [13].

This study aims to explore how sex may influence differences in therapeutic response in a real-world population previously exposed to anti-TNF agents and initiated on treatment with vedolizumab (VDZ), ustekinumab (UST), or tofacitinib (TOFA).

## 2. Materials and Methods

### 2.1. Study Design and Setting

In this multicentre retrospective observational cohort study, the medical records of patients with UC were retrospectively reviewed from 2019 to 2024 to identify and collect variables of interest. The study focused on anti-TNF-experienced patients who, within this period, had initiated treatment with biologics or tofacitinib and for whom various variables were available at treatment initiation and 8, 24, and 48 weeks thereafter. Several variables were collected, including demographics (e.g., birth-assigned sex, age, smoking status) and medical history (comorbidities, age at UC diagnosis, previous treatments undertaken), as well as clinical features such as the partial Mayo score [14] (PMS), faecal calprotectin, C-reactive protein (CRP), and erythrocyte sedimentation rate (ESR).

This study adhered to the Declaration of Helsinki (1975) and the Italian Medicines Agency’s determination of 20 March 2008. Ethics committee approval was obtained by “Brotzu” Hospital (Cagliari, Italy, PROT. PG/2022/18006). All data were strictly anonymised, analysed in aggregate form, and could not be traced back to individual patients. The manuscript was prepared according to the guidelines for the strengthening of the reporting of observational studies in epidemiology (STROBE) [15].

### 2.2. Inclusion and Exclusion Criteria

Adult patients were included if they were prescribed a biologic or small molecule with a precise diagnosis of UC and had received an indication for biologic therapy in accordance with current guidelines [16]. Patients with an uncertain diagnosis or undergoing differential diagnosis for other conditions (e.g., ischemic, infectious, or radiation colitis; microscopic or indeterminate colitis; or suspected Crohn’s disease) were excluded. Exclusion criteria also included severe conditions such as fulminant colitis, acute severe UC, toxic megacolon, major colonic surgery (e.g., subtotal or total colectomy, proctocolectomy), and gender-affirming surgery. Patients undergoing hormone therapy for gender-affirming purposes were likewise excluded from the analysis. Additionally, patients diagnosed with cancer within six months of baseline were excluded.

### 2.3. Study Outcomes

The primary aim was to assess intra-treatment differences based on assigned sex (male vs. female). The secondary aim was to evaluate intertreatment differences. Intra-treatment analysis compared outcomes between males and females within each treatment group (VDZ, UST, TOFA), while intertreatment analysis compared outcomes across the three treatments.

To this end, various clinical outcomes were assessed, including clinical remission (evaluated at 8, 24, and 48 weeks from baseline), defined as PMS ≤ 1 [17]. Steroid-free clinical remission was also evaluated at each time point, incorporating the additional criterion of the absence of steroid use at the respective time. Clinical response (assessed at 8, 24, and 48 weeks from baseline) was defined as a reduction of at least 2 points in the PMS compared to baseline [17]. Additionally, two outcomes—sustained clinical remission and sustained clinical response—were assessed, defined as the persistence of remission or response, respectively, at all three evaluated time points (i.e., 8, 24, and 48 weeks from baseline), according to the previously described criteria. Endoscopic response at 48 weeks was defined as a reduction of at least 1 point in the Mayo endoscopic score compared to baseline [17]. Endoscopic remission at the same time point was defined as a Mayo endoscopic score < 2 [17]. As previously stated, sex-based variations over the time points considered in the retrospective analysis were also assessed for various parameters (i.e., PMS, CRP, ESR, and faecal calprotectin). A final clinical evaluation was conducted to assess the outcome, stratified by sex and treatment, in patients who achieved clinical remission beyond 8 weeks (defined as late remitters). Finally, adverse events (AE) were assessed according to the WHO classification [18].

### 2.4. Statistical Analysis

Descriptive statistics were employed for data presentation, with continuous variables expressed as median (interquartile range) based on data normality, assessed using the Kolmogorov–Smirnov or Shapiro–Wilk tests. Categorical and ordinal variables were presented as frequencies, reporting the absolute counts and percentages. Qualitative non-continuous variables were compared using the Chi-square (χ^2^) test or Fisher’s exact test, as appropriate. For comparisons involving ordinal and continuous variables, the Mann–Whitney U test or the Kruskal–Wallis test was applied, depending on the degrees of freedom of the grouping variable. Bonferroni correction was applied for multiple comparisons. A logistic regression model was constructed to assess the influence of sex on target variables of interest. This model was evaluated using the Hosmer–Lemeshow goodness-of-fit test and the Cox and Snell R^2^ and Nagelkerke R^2^ values, with the data expressed as the exponential of B [i.e., exp(B)]. This was presented as the Odds Ratio (OR), with risk measures reported as the OR alongside its 95% confidence interval (95% CI).

Additionally, ORs were adjusted for potential confounding variables (aORs). Kendall’s tau-b test was applied where correlation analysis was required, reporting the correlation coefficient (τ) and the corresponding *p*-value. All analyses were conducted with a two-tailed significance level of 5% (alpha = 0.05), with statistical significance defined as a *p*-value < 0.05. Statistical analyses were performed using IBM^®^ SPSS^®^ software (version 25, IBM Corp.©, Armonk, NY, USA), while graphing was completed with Prism GraphPad^®^ (version 9.5.0, GraphPad Software LLC©, Boston, MA, USA).

## 3. Results

### 3.1. Population Characteristics

The analysis included 602 consecutive patients with a median overall age of 51 (38–61) years and a slightly higher representation of females (302, 50.2%). As shown in Table 1, most patients (260, 43.1%) were receiving VDZ, followed by UST (193, 32.05%) and TOFA (149, 24.7%). Among patients treated with VDZ, males were older than females (*p* = 0.01, Table 1). An opposite trend was observed in patients treated with UST (*p* = 0.045) and TOFA (*p* = 0.006, Table 1). Additionally, in the VDZ-treated group, males were significantly more likely to be smokers compared to females (16.1% vs. 8.1%, *p* = 0.044).

Most patients exhibited pancolonic disease (E3, 363; 60.3%), were receiving 5-ASA therapy (582; 96.7%), and were on steroids at the start of biologics/TOFA (518; 86%). The list of comorbidities recorded in the entire sample is presented in Table S1. As shown in the same table, the three most common comorbidities were hypertension (53, 8.8%), diabetes mellitus (31, 5.1%), and, finally, anxiety–depressive disorders (19, 3.2%). In addition, the primary extra-intestinal manifestation was psoriasis (11, 1.83%), followed by enteropathic spondylarthritis (9, 1.5%).

The same table also shows that the most frequent comorbidities did not differ in distribution by sex (*p* > 0.05).

### 3.2. Intra-Treatment Differences: How Sexes Respond Under the Same Treatment Conditions

The effectiveness outcomes analysed across the overall sample demonstrated homogeneity between sexes in clinical and endoscopic parameters at all evaluation time points (Table 2).

**Figure 1:**
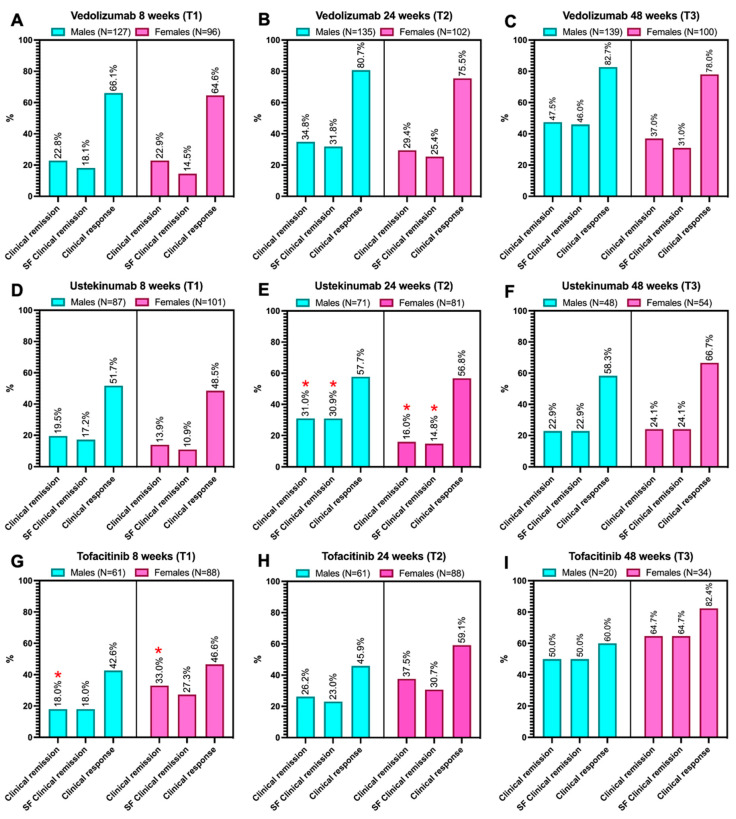
Clinical outcomes are split by sex (males vs. females) for each type of biologic (**A**–**I**). The outcomes presented include clinical remission, steroid-free (SF) clinical remission, and clinical response. The sample sizes for each subgroup are indicated at the top of each chart. Statistically significant differences (*p* < 0.05) between the two groups, if present, are marked with a red asterisk. The test evaluates potential differences between the two subgroups (males and females), with an alpha error threshold of 5%. The test employed in this setting was the χ^2^ or Fisher’s exact test, where appropriate. The inter-treatment differences in the outcomes shown in the figures are described in the article’s text.

However, when data were stratified by treatment type (biologic or tofacitinib), distinct results emerged (Figure 1). Among patients treated with UST, males showed a higher clinical remission rate at 24 weeks (31% vs. 16%, *p* = 0.029), which also extended to steroid-free remission (30.9% vs. 14.8%, *p* = 0.013) compared to females (Figure 1E). Conversely, among patients receiving TOFA, females exhibited a higher clinical remission rate at 8 weeks (33% vs. 18%, *p* = 0.043, Figure 1G). No additional sex-based differences were identified within the same treatment.

### 3.3. Inter-Treatment Differences: How Sexes Respond Across Different Treatments

During the retrospective analysis, the data demonstrated a consistent real-world effectiveness profile for VDZ clinical response in males over time (with greater pressure from TOFA at 48 weeks for remission outcomes). Conversely, in females, TOFA maintained a trend of superiority in real-life clinical remission (including steroid-free remission) across all evaluation time points, whereas VDZ remained optimal for clinical response but only for up to 48 weeks. Figure S1 compares baseline disease activity variables stratified by sex and the type of biological therapy employed.

In detail, in males, clinical response at 8 weeks was higher among patients treated with VDZ (66.1%) compared to those receiving UST (51.7%) and TOFA (42.6%), as shown in Figure 1A,D,G (*p* = 0.006). This trend persisted at 24 weeks (Figure 1B,E,H, *p* < 0.001) and at 48 weeks (Figure 1C,F,I, *p* = 0.001). However, in the 48-week comparison, TOFA slightly outperformed UST (60% vs. 58.3%). At 48 weeks, clinical remission was higher in patients treated with TOFA (50%) than in those on VDZ (47.5%) or UST (22.9%), as illustrated in Figure 1C,F,I (*p* = 0.009). This finding also extended to steroid-free remission (Figure 1C,F,I, *p* = 0.013).

In females at 8 weeks, TOFA demonstrated the highest rate of clinical remission (33%) compared with VDZ (22.9%) and UST (13.9%), as shown in Figure 1A,D,G (*p* = 0.008). A similar trend was observed for steroid-free clinical remission (Figure 1A,D,G, *p* = 0.007).

TOFA maintained this advantage for both clinical remission and steroid-free remission at 24 weeks (Figure 1B,E,H, all *p* < 0.05). However, at 24 weeks, VDZ outperformed TOFA in achieving clinical response (75.5%) compared to TOFA (59.1%) and UST (56.8%), as depicted in the exact figures (*p* = 0.013). At 48 weeks, despite differences in sample sizes across treatments, TOFA outperformed all other treatments for clinical remission (*p* = 0.001) and steroid-free clinical remission (*p* = 0.001; Figure 1C,F,I). The regression analysis did not identify specific predictive variables for response or clinical remission (steroid-free) outcomes.

### 3.4. Sustained Clinical Response and Remission: What Sex-Based Differences

As shown in Table 1, the rates of sustained clinical remission (12.4% vs. 11%) and response (40% vs. 37.3%) in the overall sample were comparable between males and females (*p* = 0.605 and *p* = 0.544, respectively).

In male patients, however, as shown in Table S2, those treated with VDZ (17.4%) demonstrated greater sustained remission than those treated with TOFA (8.6%) or UST (6.4%) across the three time points (*p* = 0.024). This was also observed for sustained clinical response, with VDZ (58.4%) showing significantly higher rates than UST and TOFA, which had notably lower rates (Table S2, *p* < 0.001). In females, this finding was reflected only for sustained clinical response, with VDZ (55.9%) significantly higher than TOFA (26.6%) and UST (24.1%) (*p* < 0.001). However, for sustained clinical remission, the three treatments were nearly equivalent (Table S2, *p* = 0.557). In contrast, the comparison analysis between males and females for the same treatment showed that the rates of sustained clinical remission or response were almost identical, mirroring what was observed in the overall sample (Table S2).

### 3.5. Late Remitters and Sex

**Figure 2:**
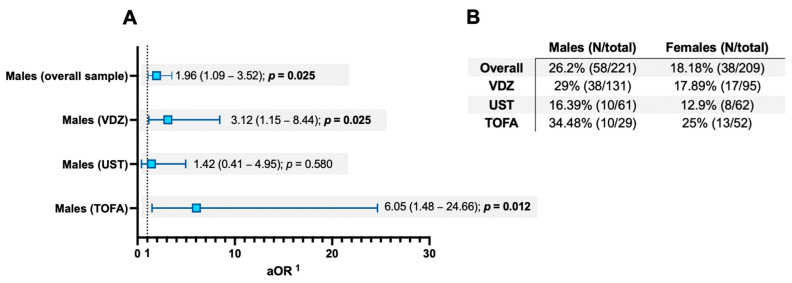
(**A**) Forest plot of a logistic regression analysis examining male sex and the likelihood of delayed remission to therapy (defined as clinical remission achieved beyond the eighth week after baseline treatment). (**B**) Crude rates of late remitters stratified by sex (i.e., males and females) across the overall sample and by the type of drug used for ulcerative colitis treatment. The table reports the rates alongside the number of late remitters over the total number of patients in each subgroup. ^1^ The odds ratio (OR) calculated is adjusted (aOR) for confounding factors (setting females as reference) such as age, disease duration, baseline partial Mayo score, baseline C-reactive protein, and baseline faecal calprotectin levels. The corresponding 95% confidence interval (CI) and computed *p*-value are also indicated in parentheses. *p*-values are two-tailed, with an alpha error of 5%, and significant values (i.e., *p* < 0.05) are highlighted in bold for easier interpretation. Acronyms: VDZ: vedolizumab; UST: ustekinumab; TOFA: tofacitinib; N: sample size. The vertical dashed line indicates an aOR = 1.

In our analysis, the rate of late remitters was significantly higher in males (26.2%) compared to females (18.18%), as shown in Figure 2 (*p* = 0.04). Additionally, as depicted in the figure, we calculated an aOR for late remission in males of 1.96 (95% CI 1.09–3.52, *p* = 0.025). This pattern of increased risk persisted, albeit within the moderate limits of sample size reduction due to data splitting, for patients treated with VDZ and TOFA when stratified by treatment type (*p* < 0.05, Figure 2).

Moreover, in Table S3, intergroup differences between male and female late remitters are compared, highlighting that the samples were markedly homogeneous with respect to baseline clinical, biochemical, and endoscopic disease activity parameters. However, a trend was observed where males were more frequently treated with VDZ than females (65.5% vs. 55.3%, *p* = 0.044).

### 3.6. Endoscopic Outcomes: Sex-Specific Differences

**Figure 3:**
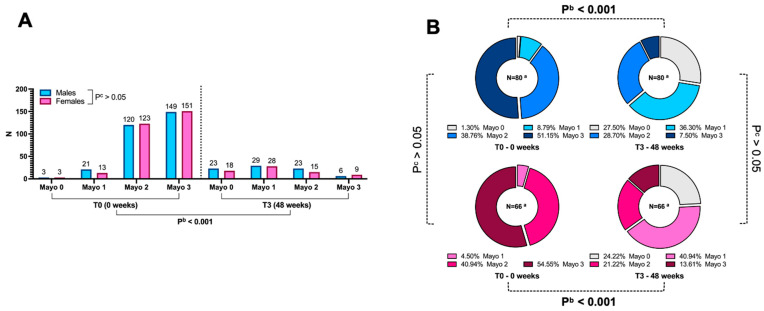
(**A**) Endoscopic disease severity (measured using the Mayo endoscopic score) at two evaluation time points (T0 and T3), stratified by sex (males and females) based on the sample size for each severity level of the score. (**B**) Sex-stratified and time-divided pie chart illustrating the relative percentages of endoscopic severity as evaluated by the Mayo endoscopic score. ^a^ N represents the global sample size on which the percentages are calculated, i.e., the number of patients with available endoscopic variable data for the specific evaluation. ^b^ The test evaluates whether the Mayo endoscopic score variation within the specified time (i.e., T0–T3) interval indicated is significant within each group (males or females), with an alpha error threshold set at 5%. Statistically significant differences (*p* < 0.05) between the two groups, if present, are marked in bold. ^c^ The test is conducted to evaluate potential endoscopic differences between the two subgroups considered (males and females), with an alpha error threshold set at 5%. Significant values (*p* < 0.05) are highlighted in bold for easier reference.

As highlighted in Figure 3, the Mayo endoscopic score (and thus the endoscopic severity of the disease) was nearly comparable between the sexes at baseline and 48 weeks. Additionally, although the T0-T3 variation was marked in both sexes (*p* < 0.001), it did not differ between them (*p* > 0.05). Treatment-specific differences were not performed due to inter-treatment sample heterogeneity.

Consequently, endoscopic outcomes, namely remission and endoscopic response at 48 weeks, were predictably indifferent by sex (*p* = 0.846 and *p* = 0.667, respectively; Table 3).

In both males and females, we investigated the strength of the correlation between patients’ main clinical and biochemical variables and endoscopic severity at both week 0 and week 48. As shown in Table S4, biochemical variables (i.e., faecal calprotectin, CRP, and ESR) and clinical variables (i.e., PMS) generally correlated positively with endoscopic severity. An interesting difference between males and females was observed in the reciprocal correlation of endoscopic severity at the two different time points, with the Mayo endoscopic score at week 0 showing a positive, albeit not extremely strong, correlation with the score at week 48 in males (τ: 0.289, *p* = 0.004), whereas in females, no correlation was observed (τ: 0.181, *p* = 0.106).

### 3.7. Sex-Specific Fluctuations of the PMS, CRP, ESR, and Faecal Calprotectin During the Retrospective Follow-Up and Adverse Events Recorded

**Figure 4:**
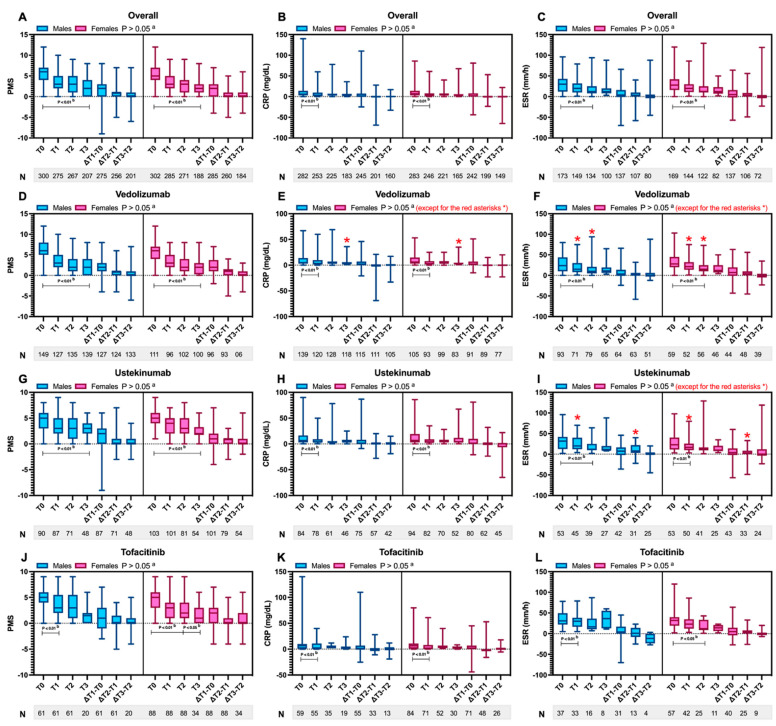
Variations in partial Mayo score (PMS), C-reactive protein (CRP), and erythrocyte sedimentation rate (ESR), stratified by sex (males vs. females) in the entire sample (**A**–**C**), among patients treated with vedolizumab (**D**–**F**), ustekinumab (**G**–**I**), and tofacitinib (**J**–**L**). For each parameter, values at baseline (T0), after 8 weeks (T1), after 24 weeks (T2), and after 48 weeks (T3) are depicted. Additionally, the differentials between the specified time points (i.e., Δ_T1−T0_, Δ_T2−T1_, and Δ_T3−T2_) are also reported. Below each graph, the sample sizes for each subgroup and time interval are also indicated. The values, being continuous variables, are reported as median and interquartile range. N: sample size. ^a^ The test evaluates whether differences exist between the two groups (males vs. females) for the value of the single parameter at the same time point analysed, with an alpha error threshold set at 5%. Statistically significant differences (*p* < 0.05) between the two groups, if present, are marked with a red asterisk. ^b^ The test evaluates whether the variation within the specified time interval indicated by the parameter bar is significant within each group (males or females), with an alpha error threshold set at 5%. Statistically significant differences (*p* < 0.05) between the two groups, if present, are marked in bold.

**Figure 5:**
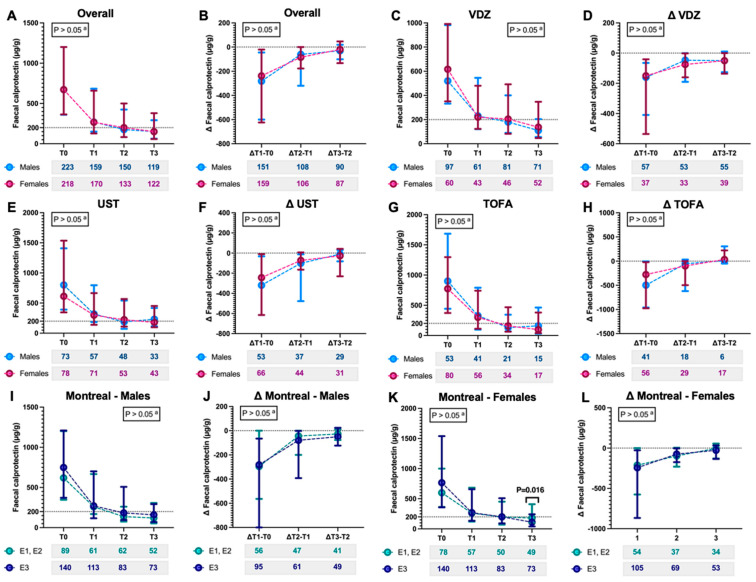
(**A**) Fluctuations in faecal calprotectin, stratified by sex (male and female), at different assessment time points: baseline (T0), after 8 weeks (T1), 24 weeks (T2), 48 weeks (T3), and (**B**) differences (Δ) between time points in patients with available data for the respective comparisons. These data are also presented for specific types of biologics/small molecules, specifically vedolizumab (**C**,**D**), ustekinumab (**E**,**F**), and tofacitinib (**G**,**H**). The evaluation was conducted in both males (**I**,**J**) and females (**K**,**L**) concerning the extent of the disease, dichotomised as proctitis or left-sided colitis (E1, E2) versus pancolitis (E3). Below each graph, the sample sizes for each subgroup and time interval are also indicated. The values, being continuous variables, are reported as median and interquartile range. ^a^ The test is conducted to evaluate potential differences at every time between the two subgroups considered (males and females), with an alpha error threshold set at 5%. Significant values (*p* < 0.05) are boldly highlighted for easier reference. Acronyms: VDZ: vedolizumab; UST: ustekinumab; TOFA: tofacitinib.

Figure 4 and Figure 5 summarise the variations in these parameters. The PMS did not show significant sex-based differences at baseline, in either the entire cohort or across treatments, including VDZ (*p* = 0.240), UST (*p* = 0.542), and TOFA (*p* = 0.589), suggesting that the sexes started with comparable levels of clinical disease activity across all treatments (Table 3). Moreover, the PMS demonstrated a consistent reduction over time, both within the overall cohort and across treatments in both sexes (*p* < 0.05; Figure 4A,D,G,J). This trend was particularly pronounced during the first 8 weeks (*p* < 0.01, Figure 4J) in males treated with TOFA, compared with other observation times (*p* > 0.05, Figure 4J). No marked sex-specific differences emerged for this parameter.

The CRP, both across the overall sample and within different treatments and sexes, showed a marked reduction, which, as expected, was most prominent during the first 8 weeks (*p* < 0.01; Figure 4B,E,H,K). After this period, the values largely remained within normal limits, with minimal fluctuations (Figure 4B,E,H,K). The only sex-specific difference observed was in patients treated with VDZ, where, at the end of the retrospective follow-up (48 weeks), CRP was slightly higher in males than in females [3 (3–6) vs. 3 (3–5), *p* = 0.021; Figure 4E].

Similarly to CRP, ESR showed a reduction from baseline to week 48 in the overall population, in both sexes, and across all treatments considered (*p* < 0.05; Figure 4C,F,I,L). However, the change was significant only in females treated with UST within the first 8 weeks (*p* < 0.01; Figure 4I). In contrast to CRP, however, many more treatment-specific and sex-specific differences emerged in ESR. In patients treated with VDZ at 8 weeks [21.5 (13.5–32.5) vs. 15 (8–30) mm/h, *p* = 0.025] and at 24 weeks [15 (10–25) vs. 10 (5–21) mm/h, *p* = 0.046], females had higher ESR levels (Figure 4F). In patients treated with UST, this sex-specific difference was more pronounced in males compared to females at 8 weeks [20 (12–40) vs. 16.5 (10–25.75) mm/h, *p* = 0.036] and in the Δ_T2−T1_ [8 (3–22) vs. 3 (0–8.5), *p* = 0.015], as shown in Figure 4I. No sex-specific differences emerged for patients treated with TOFA.

Finally, for faecal calprotectin, as shown in Figure 5, the temporal variations were broadly comparable between males and females, regardless of treatment or disease localisation (*p* > 0.05). In both sexes, the temporal variations were negative, with a reduction in faecal calprotectin levels (*p* < 0.01; Figure 5), except in females during the interval between 24 and 48 weeks (*p* = 0.09). Moreover, even when comparing between sexes (and between treatments), the variations (Δ) in calprotectin levels throughout the follow-up did not show significant differences (*p* > 0.05; Figure 5). The changes in steroid use across the evaluation time points are detailed in Table S5. Information regarding any potential AEs is provided in Table S6. Regarding safety, no serious adverse events were recorded, and the main adverse event was hypersensitivity reactions, which were observed in only three cases (0.5%). As for infections due to Herpes Zoster, there were two cases (0.33%). Overall, they were so few (Table S6) that stratified analyses by sex were not feasible.

## 4. Discussion

This retrospective analysis demonstrated specific sex-based differences in bio-experienced UC patients previously treated with anti-TNF agents and subsequently receiving VDZ, UST, or TOFA. Most of the available evidence has focused on anti-TNF therapies (13). Some studies have highlighted that male patients treated with anti-TNF agents (infliximab or adalimumab) had a higher risk of experiencing reduced trough drug levels in the initial weeks of treatment compared to females [19]. This variation was partially explained by specific polymorphisms in genes regulating IgG half-life [19]. In general, however, sex has not shown significant differential associations with therapeutic outcomes in UC patients treated with anti-TNF agents in most studies [20]. Although strong associations are lacking, male sex appears to be at a higher risk of loss of response to anti-TNF therapy [21]. Similar comparisons cannot be drawn from our data, considering that our entire cohort was uniformly anti-TNF-exposed.

Our data indicate that male patients treated with UST exhibited better intra-treatment therapeutic performance than females. Thunberg et al. [22], in a study of 133 UC patients, mostly bio-experienced, found that male sex was associated with greater UST persistence at 16 weeks (aOR: 4, 95% CI 1.35–11.83). Another smaller study (≈60 IBD patients, 19.6% with UC) suggested that females (OR 4.56) were more likely to achieve stable UST trough levels [23]. However, the latter data were obtained from a small population, and a significant difference in fat mass between males and females was observed, which prevented a firm conclusion. Conversely, a more extensive study involving 620 UC patients treated with UST found that male sex (OR: 0.5, 95% CI 0.4–0.8) was associated with a lower likelihood of achieving steroid-free remission at week 16 of treatment [24]. In a previous real-world study on UST in UC, no significant differences in therapeutic outcomes were observed by us with sex [25].

An inter-treatment comparison in males revealed a marked superiority of VDZ at all retrospective observation time points, with higher clinical response rates than UST and TOFA. Chhibba et al. [26], in a post hoc analysis of the VARSITY trial [27] comparing VDZ and adalimumab in UC, found that at week 6, females were more likely to achieve endoscopic improvement and had higher VDZ trough levels. However, no differences in clinical outcomes were observed through week 52. However, this population is not directly comparable to ours, as fewer than 25% had prior anti-TNF exposure. Eriksson et al. [28] reported no significant differences in efficacy between sexes in a real-life Swedish cohort of 246 IBD patients (37.3% with UC) treated with VDZ, the vast majority of whom were anti-TNF-exposed. However, they observed poorer performance in females, with an aHR of 2.75 for discontinuation of VDZ due to intolerance.

Among females, TOFA showed superior clinical remission rates along the study timeline compared to other treatments. Despite the challenges in finding comparable studies, Vong et al. [29] conducted a thorough pharmacokinetic study based on data from TOFA phase 2 and 3 registrational randomised controlled trials. Findings showed that females had a 13.2% lower apparent oral clearance and a 15.5% lower apparent distribution volume than males, though these differences do not justify sex-based dose adjustments.

In any case, it is noteworthy that data elucidating the subtle pharmacokinetic differences between males and females with respect to biologic agents and small molecules in IBD are notably lacking, which would be essential to assess whether such differences might have real clinical relevance and thus help guide more targeted and sex-personalised prescribing decisions.

Our data also showed a higher likelihood of delayed remission among males than among females, with an aOR of 1.958 (95% CI 1.088–3.524). In profiling late remitters, a post hoc analysis of the registrational UNIFI [30] trial suggests that delayed responders treated with UST exhibit a higher pre-treatment inflammatory burden. However, they achieve outcomes similar to those of early responders one year after treatment [31]. In the context of TOFA, Sandborn et al. [32], using data from the OCTAVE [33] trials, compared the drug’s efficacy between delayed responders and those requiring an extended induction period of 16 weeks instead of 8 weeks. Their findings demonstrated that delayed responders could maintain remission in most cases, even up to three years after treatment. In addition, Narula et al. [34] reported in a post hoc analysis of data from the SEAVUE clinical trial programme that patients with moderate to severe CD treated with adalimumab or UST, both early and delayed responders (defined as those responding at week 16 after non-response at week 8), had similar one-year outcomes with both drugs.

However, none of these analyses have documented sex-based differences in delayed response or remission rates. Nevertheless, given this, the finding of delayed remission may have clinical interest, as outcomes, while requiring further supporting evidence, can often be comparable to those of early responders. Therefore, identifying variables (in this case, sex based) that may support the continuation of a treatment, rather than prematurely discarding therapeutic options based on an initial nonresponse, is crucial.

Additionally, our data show comparable faecal calprotectin trajectories in males and females (Figure 5). Therefore, it is not surprising that endoscopic outcomes also showed no sex-based differences. However, a weak positive correlation between baseline endoscopic severity and that at 48 weeks was observed only in males (τ = 0.289, *p* < 0.001).

These findings highlight the need for greater awareness of sex-based outcomes in IBD clinical trials. Such comparisons are often overlooked or conducted with significant heterogeneity, underscoring the need for further research to identify parameters genuinely influenced by sex and their impact on therapeutic outcomes. Integrating sex-based knowledge in IBD research has been proposed to improve data interpretation and validity [13], given the well-established differences in epidemiology, pathogenesis, genetics, and disease phenotype [2]. Assuming these differences do not affect therapeutic outcomes is unwarranted.

This study has several limitations, including its retrospective design and the reduced sample size at later time points, which affect the statistical power of the analyses and strengthen conclusions at earlier stages. This introduces a potential selection and causal inference bias inherent to the retrospective design.

Additionally, some data are missing for certain variables (e.g., endoscopic parameters), reflecting the dataset’s real-world nature. Therefore, these data show greater weakness at treatment time points (i.e., long-term outcomes) further from the inductive baseline (e.g., 48 weeks), as indicated by lower statistical power.

However, its strengths include a large multicentre dataset, a balanced male-to-female distribution, and baseline pre-treatment homogeneity in inflammatory burden and disease severity, which enhance the reliability of time-dependent comparisons. A further limitation of the study is the absence of a differential analysis of menopausal status among the included patients in the female cohort. However, this would have been difficult to perform in an observational, real-world setting, where assessing hormonal status (e.g., FSH measurement) to help diagnose menopause is not part of standard management for UC patients. The lack of hormonal evaluations also precludes pathophysiological insights into these differences, which, however, lie beyond the scope, feasibility, and outcomes of this work. Finally, in almost all Italian regions, first-line prescription of advanced medical therapy (biologics and small molecules) for UC patients is generally required (except in specific cases with justified contraindications) to involve the use of an anti-TNF biosimilar for pharmacoeconomic reasons. This explains why, given the real-world nature of this study, it would have been unrealistic to select a subgroup of patients naïve to anti-TNF therapy and treated de novo with VDZ, UST, or TOFA. Conversely, since the outcomes of this analysis were fundamentally oriented towards a sex-based evaluation of advanced medical therapy, the inclusion of a biologic-naïve comparison group was not considered appropriate, as such patients, according to international guidelines, remain subject to a step-up treatment protocol that still involves conventional therapy as the first-line option, rendering such a comparison unbalanced and uninformative for this study.

In any case, notwithstanding these considerations, which are unmodifiable and independent of the study design, this limits the applicability of these data to a bio-naïve population.

Finally, this study, being real-world in nature, could not include detailed pharmacokinetic assessments, including those adjusted for body weight, which is a variable influencing such parameters. Therefore, future sex-based studies should address this aspect.

## 5. Conclusions

In conclusion, these data suggest sex-based differences in therapeutic response among anti-TNF-exposed UC patients receiving VDZ, UST, or TOFA as subsequent-line treatment. Further research is needed to elucidate the underlying mechanisms.

## Figures and Tables

**Table 1:** Clinical and demographic differences at baseline between the subgroups (males and females) considered, stratified by treatment.

Parameter	VDZ (N = 260)		*p*-Value ^1^	UST (N = 193)		*p*-Value ^1^	TOFA (N = 149)		*p*-Value ^1^
	M (N = 149)	F (N = 111)		M (N = 90)	F (N = 103)		M (N = 61)	F (N = 88)	
Age	55 (44.5–65.5)	51 (42–60)	**0.01**	47 (31–63)	54 (40–65)	**0.045**	42 (30–49.5)	48.5 (35–58)	**0.006**
Smoking status									
Active	24 (16.1%)	9 (8.1%)	**0.044**	7 (7.8%)	8 (7.8%)	0.119	10 (13.1%)	11 (12.5%)	0.110
Not smoker	105 (70.5%)	91 (82%)		11 (12.2%)	22 (21.4%)		8 (13.1%)	24 (27.3%)	
Former smoker	20 (13.4%)	11 (9.9%)		72 (80%)	73 (70.9%)		43 (70.5%)	53 (60.2%)	
Appendectomy (yes)	15 (10.1%)	7 (6.3%)	0.369 ^4^	7 (7.8%)	6 (5.8%)	0.775 ^4^	1 (1.6%)	3 (3.4%)	0.645 ^4^
UC duration (years)	10 (4.5–16.5)	11 (7–18)	0.404 ^4^	9 (5.75–15.5)	9 (5–16)	0.806	9 (5–15.5)	8 (4–15)	0.496 ^4^
Montreal classification									
E1 (proctitis)	2 (1.3%)	3 (2.7%)	0.411	2 (2.2%)	0 (0%)	0.148	2 (3.3%)	4 (4.5%)	0.973
E2 (left colitis)	63 (42.3%)	39 (35.1%)		38 (42.2%)	36 (35%)		21 (34.4%)	29 (33%)	
E3 (pancolitis)	84 (56.4%)	69 (62.2%)		50 (55.6%)	67 (65%)		38 (62.3%)	55 (62.5%)	
Previous treatments ^2^									
Infliximab	107 (71.8%)	81 (73%)	0.836 ^4^	69 (76.7%)	81 (78.6%)	0.742 ^4^	56 (91.8%)	68 (77.3%)	**0.025 ^4^**
Adalimumab	53 (35.6%)	36 (32.4%)	0.598 ^4^	42 (46.7%)	37 (35.9%)	0.130 ^4^	26 (42.6%)	41 (46.6%)	0.738 ^4^
Golimumab	46 (30.9%)	32 (28.8%)	0.722 ^4^	6 (6.7%)	13 (12.6%)	0.166 ^4^	14 (23%)	16 (18.2%)	0.536 ^4^
Vedolizumab	0 (0%)	0 (0%)	N.A. ^5^	50 (55.6%)	55 (53.4%)	0.764 ^4^	37 (60.7%)	42 (47.7%)	0.321 ^4^
Ustekinumab	3 (2%)	0 (0%)	0.263 ^4^	0 (0%)	0 (0%)	N.A. ^5^	7 (11.5%)	9 (10.2%)	0.795 ^4^
5-ASA (yes)	140 (94%)	107 (96.4%)	0.567 ^4^	90 (100%)	102 (99%)	0.999 ^4^	58 (95.1%)	85 (96.6%)	0.689 ^4^
Steroids (yes)	130 (87.2%)	103 (92.8%)	0.158 ^4^	84 (93.3%)	98 (95.1%)	0.758 ^4^	44 (72.1%)	59 (67%)	0.590 ^4^
Thiopurines ^3^ (yes)	71 (47.6%)	45 (40.5%)	0.260 ^4^	40 (44.4%)	27 (26.2%)	**0.008 ^4^**	18 (29.5%)	24 (27.3%)	0.854 ^4^
Methotrexate ^3^ (yes)	15 (10.06%)	12 (10.8%)	0.840 ^4^	4 (4.4%)	3 (2.9%)	0.570 ^4^	7 (11.5%)	4 (4.5%)	0.125 ^4^
Latent TBC (yes)	8 (5.4%)	3 (2.7%)	0.362 ^4^	0 (0%)	6 (5.8%)	**0.031 ^4^**	1 (1.6%)	1 (1.1%)	0.999 ^4^
HBV infection (yes)	0 (0%)	0 (0%)	N.A. ^5^	0 (0%)	4 (3.9%)	0.125 ^4^	1 (1.6%)	0 (0%)	0.409 ^4^

Continuous variables are presented as median (interquartile range), while categorical or ordinal variables are expressed as frequencies, i.e., counts (percentage of the total in the subgroup considered, male or female). Acronyms: N: sample size; VDZ: vedolizumab; UST: ustekinumab; TOFA: tofacitinib; M: males; F: females; N.A.: not applicable; 5-ASA: 5-aminosalicylic acid; HBV: hepatitis B virus; TBC: tuberculosis. Notes: ^1^ The test evaluates potential differences between the two subgroups considered (males and females), with an alpha error threshold set at 5%. Significant values (*p* < 0.05) are boldly highlighted for easier reference. ^2^ The percentage in this row is calculated based on the total number of patients in the respective subgroup (males or females) who underwent the specified treatment. Consequently, the count also includes patients who underwent more than one treatment among the considered options. ^3^ In this row, the data refer to prior use of thiopurines or methotrexate, not their active use at baseline. ^4^ The test employed in this setting was the χ^2^ or Fisher’s exact test, where appropriate. ^5^ In this instance, the test was not performed due to the absence of cases in the subgroups or if the two groups had exactly equal percentages.

**Table 2:** Overall clinical and endoscopic outcomes stratified by the two subgroups of interest (males and females).

Parameter ^1^	Males (N) ^2^	Females (N) ^2^	*p*-Value ^3^
Clinical remission 8 weeks	20.7% (275)	22.8% (285)	0.551
Steroid-free ^4^	17.8%	17.1%	0.924
Clinical response 8 weeks	56.4% (275)	55.4% (285)	0.826
Clinical remission 24 weeks	31.8% (267)	28% (271)	0.337
Steroid-free ^4^	29.5%	23.9%	0.132
Clinical response 24 weeks	66.7% (267)	64.6% (271)	0.610
Clinical remission 48 weeks	42% (207)	38.3% (188)	0.450
Steroid-free ^4^	41%	35.1%	0.301
Clinical response 48 weeks	74.9% (207)	75.5% (188)	0.881
Sustained clinical remission	12.4% (274)	11% (273)	0.605
Sustained clinical response	40% (240)	37.3% (236)	0.544
Endoscopic remission 48 weeks	64.2% (81)	65.7% (70)	0.846
Endoscopic response 48 weeks	78.8% (80)	75.8% (66)	0.667

Acronym: N: sample size. Notes: ^1^ The time points considered for calculating the variable are as follows: at 8 weeks, 24 weeks, and 48 weeks. ^2^ In parentheses, the sample size used to calculate the data is indicated, representing the number of subjects with evaluations available at both time points to compute the differential necessary for identifying the outcome of interest. ^3^ The test evaluates potential differences between the two subgroups considered (males and females), with an alpha error threshold set at 5%. The test employed in this setting was the χ^2^ or Fisher’s exact test, where appropriate. ^4^ The clinical remission parameter over time is also calculated as the steroid-free percentage, i.e., patients recorded as being in clinical remission and a declared absence of steroid use.

**Table 3:** Baseline (T0) disease activity parameters were analysed in the global retrospective cohort and stratified by treatment type: biologics (vedolizumab, ustekinumab) and small molecules (tofacitinib).

Parameter	Males [N] ^1^	Females [N] ^1^	*p*-Value ^2^
Overall sample			
PMS	6 (4–7) [300]	5 (4–7) [302]	0.167
Faecal calprotectin (μg/g)	671 (360–1200) [233]	672.5 (364.5–1200) [218]	0.882
CRP (mg/dL)	6 (3–14.07) [282]	6.8 (3–14.3) [283]	0.729
Mayo endoscopic score	3 (2–3) [293]	3 (2–3) [290]	0.573
ESR (mm/h)	30 (12–43) [173]	28 (16–42) [169]	0.738
Vedolizumab			
PMS	6 (5–8) [149]	6 (4–7) [111]	0.240
Faecal calprotectin (μg/g)	520 (332–982) [97]	617 (350.5–991.25) [60]	0.484
CRP (mg/dL)	5 (3–13.9) [139]	6 (3–15) [105]	0.725
Mayo endoscopic score	3 (2–3) [144]	2 (2–3) [103]	0.630
ESR (mm/h)	24 (10–44) [83]	28 (20–45) [59]	0.099
Ustekinumab			
PMS	5 (3–6) [90]	5 (4–6) [103]	0.542
Faecal calprotectin (μg/g)	800 (391.5–1410) [73]	612.5 (346–1535.5) [78]	0.784
CRP (mg/dL)	6.5 (3–16.5) [84]	6.8 (3–19.25) [94]	0.342
Mayo endoscopic score	2 (2–3) [89]	3 (2–3) [101]	0.093
ESR (mm/h)	32 (12.5–42) [53]	23 (11.5–40.5) [53]	0.450
Tofacitinib			
PMS	5 (4–6) [61]	5 (3–6) [88]	0.589
Faecal calprotectin (μg/g)	900 (443–1685) [53]	773.5 (372–1297.5) [80]	0.314
CRP (mg/dL)	7 (1–10) [59]	6.5 (0.99–10.97) [84]	0.556
Mayo endoscopic score	3 (2–3) [60]	3 (2–3) [86]	0.529
ESR (mm/h)	31 (22–49.5) [37]	32 (19–40) [57]	0.407

Continuous variables are presented as median (interquartile range), while categorical or ordinal variables are expressed as frequencies, i.e., counts (percentage of the total in the subgroup considered, male or female). Acronyms: N: sample size; PMS: partial Mayo score; CRP: C-reactive protein; ESR: erythrocyte sedimentation rate. Notes: ^1^ In parentheses, the sample size used to calculate the data is indicated, representing the number of subjects with evaluations available. ^2^ The test evaluates potential differences between the two subgroups considered (males and females), with an alpha error threshold set at 5%.

## Data Availability

The original contributions presented in the study are included in the article; further inquiries can be directed to the corresponding author.

## Supplementary Materials

The following supporting information can be downloaded at https://www.mdpi.com/article/10.3390/jcm14217476/s1, Figure S1: Levels of partial Mayo score (PMS, A), faecal calprotectin (B), C-reactive protein (CRP, C), Mayo endoscopic score (D), and erythrocyte sedimentation rate (ESR, E) at baseline (T0) in the overall sample, stratified by sex (males and females). The variables are presented as median (interquartile range). The test evaluates potential differences between the two subgroups considered (males and females), with an alpha error threshold set at 5%. Significant values (*p* < 0.05) are boldly highlighted for easier reference. The sample size of each subgroup can be deduced from Table 2; Table S1: List of comorbidities recorded in the sample in descending order of frequency; Table S2: Sex-based differences in sustained clinical remission and response concerning the type of advanced medical agent used; Table S3: Clinical and demographic differences at baseline between males and females late remitters; Table S4: Sex-based correlation analysis among the main clinical–demographic variables and the endoscopic disease severity at baseline and the end of the retrospective timeframe; Table S5: Steroid use stratified by sex (males and females) at the four time points where the data are available: baseline (T0), 8 weeks (T1), 24 weeks (T2), and 48 weeks (T3); Table S6: Adverse events (AEs) recorded in the retrospective follow-up of patients included in the analysis in descending order of frequency.

## Appendix A

**Italian Network for Inflammatory Bowel Diseases (IN-IBD) (Collaborators)**: **Davide G. Ribaldone**: IBD Unit, A.O.U. Citta della Salute e della Scienza”, Torino; **Antonio Ferronato**: UOSD di Endoscopia Digestiva, ULSS7 Pedemontana, Ospedale di Santorso, Santorso (VI); **Brigida Barberio**, **Greta Lorenzon**, **Caterina De Barba**, **Fabiana Zingone**: UOC di Gastroenterologia, Dipartimento di Scienze Chirurgiche, Oncologiche e Gastroenterologiche, Università di Padova, Azienda Ospedaliero-Universitaria “Sant’Antonio”, Padova; **Davide Checchin**: UOC di Gastroenterologia, Ospedale” S Giovanni e Paolo”, Mestre—Venezia; **Carla Felice**: UOC di Medicina Interna, Ospedale Universitario “Ca’ Foncello”, Treviso; **Giovanni Cataletti**: UOC di Gastroenterologia, A.O. “L. Sacco”, Milano; **Giorgia Bodini, Andrea Pasta**: UOC di Gastroenterologia, Dipartimento di Medicina Interna, IRCCS Ospedale “San Martino” Hospital, Università di Genova, Genova; **Giovanni Aragona, Patrizia Perazzo**: UOC di Gastroenterologia, Ospedale “Guglielmo da Saliceto”, Piacenza; **Federica Gaiani, Stefano Kayali**: UOC di Gastroenterologia ed Endoscopia Digestiva, Dipartimento di Medicina e Chirurgia, Ospedale Maggiore, Università di Parma, Parma; **Fabio Cortellini**: UOC di Gastroenterologia ed Endoscopia Digestiva, Ospedale” Infermi”, Rimini; **Francesco Costa**: UOC di Gastroenterologia, Azienda Ospedaliero-Universitaria Pisana, Pisa; **Lorenzo Bertani**: UOC di Gastroenterologia, Ospedale “Felice Lotti”, Azienda USL Toscana Nord Ovest, Pontedera (PI); **Antonella Scarcelli**, **Mariaelena Serio**: UOC di Gastroenterologia, Ospedale “San Salvatore”, Pesaro; **Emanuele Bendia**, **Laura Bolognini**: UOC di Gastroenterologia, Endoscopia Digestiva e Malattia infiammatorie Croniche Intestinali, Ospedale “Torrette”, Ancona; **Simona Piergallini**: UOC di Gastroenterologia, IBD Unit, Ospedale “A. Murri”, Fermo; **Maria Pina Dore**: UOC di Gastroenterologia, Azienda Ospedaliero-Universitaria “San Pietro”, Sassari; **Chiara Rocchi**: UOC di Gastroenterologia ed Endoscopia Digestiva, Ospedale “Mater Olbia” Hospital, Olbia (SS); **Francesca Onidi**, **Paolo Usai Satta**: UOC di Gastroenterologia, Azienda Ospedaliera di Rilievo Nazionale “Brotzu”, Cagliari; **Raffaele Colucci**: UOSD di Endoscopia Digestiva, Ospedale “San Matteo degli Infermi”, Spoleto (PG); **Gabrio Bassotti**, **Elisabetta Antonelli**: Sezione di Gastroenterologia ed Epatologia, Dipartimento di Medicina, Ospedale “Santa Maria della Misericordia”, Perugia; **Costantino Zampaletta**, **Giulia Rocco**, **Carlotta Sacchi**: UOC di Gastroenterologia, Ospedale “Belcolle”, Viterbo; **Franco Scaldaferri**, **Daniele Napolitano**: UOC di Medicina Interna e Gastroenterologia, Dipartimento di Scienze Mediche e Chirurgiche, IRCCS Fondazione “Policlinico Gemelli, Università Cattolica del Sacro Cuore, Roma; **Roberto Faggiani**, **Rita Monterubbianesi**: UOC di Gastroenterologia ed Endoscopia Digestiva, A.O. “S. Camillo-Forlanini”, Roma; **Cristiano Pagnini**, **Maria Giovanna Graziani**, **Maria Carla Di Paolo**: UOC di Gastroenterologia ed Endoscopia Digestiva, A.O. “S. Giovanni—Addolorata”, Roma; **Roberta Pica**, **Maddalena Zippi**, **Andrea Cocco**, **Claudio Cassieri**: UOC di Gastroenterologia ed Endoscopia Digestiva, Ospedale “S. Pertini”, Roma; **Roberto Lorenzetti**: UOC di Gastroenterologia, Ospedale Territoriale “Nuovo Regina Margherita”, Roma; **Gian Marco Giorgetti**, **Valeria Clemente**: UOSD di Endoscopia Digestiva Nutrizionale, Ospedale “S. Eugenio”, Roma; **Michela Di Fonzo**, **Federico Iacopini**: UOC di Gastroenterologia ed Endoscopia Digestiva, “Ospedale dei Castelli”, Ariccia (Roma); **Giacomo Forti**: UOSD di Endoscopia Digestiva, Ospedale “S. Maria Goretti”, Latina; **Laurino Grossi**: UOC di Gastroenterologia, Ospedale “Spirito Santo”, Università “G d’Annunzio”, Pescara; **Serafina Fiorella**: UOSD di Gastroenterologia ed Endoscopia Digestiva, Ospedale “P. Pio”, Vasto (CH); **Giovanni Lombardi**, **Marta Patturelli**: UOC di Gastroenterologia, Azienda Ospedaliera di Rilievo Nazionale “Antonio Cardarelli”, Napoli; **Giuliana Vespere**, **Silvia Sedda**, **Vittorio D’Onofrio**, **Leonardo De Luca**: UOC di Gastroenterologia, “Ospedale del Mare” ASL NA1 Centro, Napoli; **Caterina Mucherino**, **Elvira D’Antonio**, **Laura Montesano**: UOC di Gastroenterologia, Azienda Ospedaliera “S. Anna e S. Sebastiano”, Caserta; **Pietro Capone**, **Guido Daniele Villani**: UOC di Gastroenterologia ed Endoscopia Digestiva, Ospedale “T. Maresca”, Torre del Greco (NA); **Antonio Cuomo**, **Laura Donnarumma**: UOSD di Gastroenterologia, Ospedale “Umberto I”, Nocera Inferiore (SA); **Nicola Della Valle**: UOC di Gastroenterologia, A.O. “Ospedali Riuniti”, Foggia; **Giuseppe Pranzo**: UOS di Endoscopia Digestiva, Ospedale “Valle D’Itria”, Martina Franca (TA); **Paolo Tonti**, **Viviana Neve**: UOSD di Gastroenterologia ed Endoscopia Digestiva, Ospedale “A. Perrino”, Brindisi; **Libera Fanigliulo**: UOC di Gastroenterologia, A.O. “S.S. Annunziata”, Taranto; **Leonardo Allegretta**: UOC di Gastroenterologia ed Endoscopia Digestiva, Ospedale “Santa Caterina Novella”, Galatina (LE); **Manuela Marzo**: UOC di Gastroenterologia ed Endoscopia Digestiva, Ospedale “Veris-Delli Ponti”, Scorrano (LE); **Ileana Luppino**: UOC di Gastroenterologia ed Endoscopia Digestiva, Ospedale “Annunziata”, Cosenza; **Francesco Luzza**, **Rocco Spagnuolo**: Dipartimento di Scienze Mediche, UOC di Fisiopatologia Digestiva, Azienda Ospedaliera “Mater Domini”, Università di Catanzaro, Catanzaro; **Stefano Rodinò**, **Ladislava Sebkova**: UOC di Gastroenterologia, A.O. “Ciaccio-Pugliese”, Catanzaro; **Antonio De Medici**: Servizio di Gastroenterologia Territoriale, AST Catanzaro, Catanzaro Lido; **Domenico Catarella**, **Dario D’Agostino**, **Elisabetta Di Bartolo**: UOC di Gastroenterologia, ARNAS “Garibaldi”, Catania.
