# Supplementary material for: Sex-Specific Real-Life Profiling in Vedolizumab, Ustekinumab, and Tofacitinib Effectiveness in Ulcerative Colitis
Source: J Clin Med. 2025 Oct 22;14(21):7476. doi: 10.3390/jcm14217476 (PMC12609951; doi:10.3390/jcm14217476)
Supplement: Supplementary file 1 [file jcm-14-07476-s001.zip › jcm-3926171-supplementary.pdf]

SUPPLEMENTARY MATERIALS

Sex-specific real-life profiling in vedolizumab, ustekinumab, and tofacitinib effectiveness in ulcerative colitis

Supplementary Table S1. List of comorbidities recorded in the sample in descending order of frequency.

Comorbidity [1]	Frequency	Percentage (%) over the total sample (N=602)
Arterial hypertension [2]	53	8.8
Diabetes mellitus [2]	31	5.1
Anxiety-depressive syndromes [2]	19	3.2
Osteoporosis/osteopenia	18	2.99
Dyslipidaemia	17	2.8
Benign prostatic hyperplasia	13	2.16
Bronchial asthma	11	1.83
**Psoriasis**	**11**	**1.83**
Hepatic steatosis	9	1.5
**Enteropathic spondylarthritis**	**9**	**1.5**
Osteoarthritis	7	1.16
Hypothyroidism	6	1
Chronic ischaemic heart disease	6	1
Urinary calculi	5	0.83
Biliary sludge/gallbladder lithiasis	5	0.83
Hashimoto's thyroiditis	5	0.83
Fibromyalgia	5	0.83
**Primary sclerosing cholangitis**	**5**	**0.83**
Colonic diverticulosis	4	0.66
**Hidradenitis suppurativa (*acne inversa*)**	**4**	**0.66**
Bronchiectasis	4	0.66
History of breast cancer	4	0.66
Atrial fibrillation	4	0.66
**Peripheral enteropathic arthritis**	**4**	**0.66**
Thyroidectomy	4	0.66
**Pyoderma gangrenosum**	**4**	**0.66**
Chronic kidney failure	3	0.5
**Ankylosing spondylarthritis**	**3**	**0.5**
Pituitary adenoma	3	0.5
Thalassaemic trait	3	0.5
Benign breast cysts	3	0.5
Benign thyroid nodules	3	0.5
**Erythema nodosum**	**3**	**0.5**
History of renal cancer	3	0.5
**Psoriatic arthritis**	**3**	**0.5**
Migraine/headache	3	0.5
History of pulmonary thromboembolism	3	0.5
Polymyalgia rheumatica	2	0.33
Adrenal insufficiency	2	0.33
Thrombocytopenia	2	0.33
Polycystic ovary syndrome (PCOS)	2	0.33
Cholecystectomy	2	0.33
Dilated cardiomyopathy	2	0.33
Autoimmune haemolytic anaemia	2	0.33
Deep vein thrombosis (DVT)	2	0.33
Facial nerve paralysis	2	0.33
Eradicated hepatitis C virus (HCV)	2	0.33
Coeliac disease	2	0.33
Hyperthyroidism	2	0.33
Herniated disc	2	0.33
History of bladder cancer	2	0.33
Inguinal hernia	2	0.33

Pulmonary fibrosis	2	0.33
Uterine fibromatosis	2	0.33
Lichen planus	2	0.33
History of acute myocardial infarction	2	0.33
History of basal cell carcinoma	2	0.33
Splenectomy	2	0.33
History of cervical cancer	2	0.33
Enterocystoplasty	1	0.17
Pelvis-ureteric junction obstruction	1	0.17
Gastro-oesophageal reflux disease (GORD)	1	0.17
Hearing loss (hypoacusis)	1	0.17
Chronic tenosynovitis	1	0.17
Chiari malformation	1	0.17
Takotsubo syndrome	1	0.17
Gullo syndrome	1	0.17
Intraductal papillary mucinous neoplasm (IPMN)	1	0.17
Polyclonal hypergammaglobulinemia	1	0.17
Non-specific arthralgia	1	0.17
History of uterine cancer	1	0.17
Hypertrophic cardiomyopathy	1	0.17
Stable chronic subdural haematoma	1	0.17
Food IgE hypersensitivity	1	0.17
**Sweet's syndrome**	**1**	**0.17**
History of Hodgkin's lymphoma	1	0.17
Gouty arthritis	1	0.17
Tetraplegia from spinal cord injury	1	0.17
Hepatic haemangioma	1	0.17
History of ovarian cancer	1	0.17
Focal nodular hyperplasia	1	0.17
Right hemicolectomy for high-grade dysplasia in polyp	1	0.17
Meniscal pathology	1	0.17
Cerebral vasculitis	1	0.17
Simple hepatic cyst	1	0.17
History of melanoma	1	0.17
History of ischaemic stroke	1	0.17
Obstructive sleep apnoea syndrome (OSAS)	1	0.17
Chronic liver disease related to hepatitis B virus (HBV)	1	0.17
Alopecia	1	0.17
**Rheumatoid arthritis**	**1**	**0.17**
**Seronegative arthritis**	**1**	**0.17**
Stable aortic aneurysm	1	0.17
Autoimmune pericarditis	1	0.17
Monoclonal gammopathy of undetermined significance (MGUS)	1	0.17
History of transient ischaemic attack (TIA)	1	0.17
Lumbar hernia	1	0.17
Chronic pancreatitis	1	0.17
History of testicular cancer	1	0.17
Stickler syndrome	1	0.17
Non-specific vasculitis	1	0.17
Eosinophilic gastroenteritis	1	0.17
Cataract	1	0.17
Factor V Leiden mutation	1	0.17
**Uveitis**	**1**	**0.17**
Coloboma with blindness	1	0.17
**Episcleritis**	**1**	**0.17**
Complex renal cyst	1	0.17
Pilonidal cyst	1	0.17

Chronic myeloid leukaemia	1	0.17
Biological prosthesis of the mitral valve	1	0.17
Lower limb neuropathy	1	0.17
Renal artery thrombosis	1	0.17
Pancreas divisum	1	0.17
History of vagal schwannoma	1	0.17
Carpal tunnel syndrome	1	0.17
Erysipelas	1	0.17
Turbinate hypertrophy	1	0.17
History of *Helicobacter pylori* infection	1	0.17
History of *Clostridioides difficile* colitis	1	0.17
Factor VII and X deficiency	1	0.17
History of testicular torsion	1	0.17

[1] The comorbidities classified as classical extraintestinal manifestations, per the latest European Crohn's and Colitis Organisation (ECCO) Guidelines on Extraintestinal Manifestations in Inflammatory Bowel Disease (Journal of Crohn's and Colitis, 2024, 18, 1–37 [1]), are identified in bold for ease of reference.

[2] For comorbidities with a frequency ≥ 3%, an explorative comparison between males and females was conducted to evaluate potential differences using Fisher's exact test. However, no statistical significance was observed, with the alpha error set at 5% and a two-tailed P-value consistently > 0.05 in the comparisons performed.

Supplementary reference

1. Gordon, H.; Burisch, J.; Ellul, P.; Karmiris, K.; Katsanos, K.; Allocca, M.; Bamias, G.; Barreiro-de Acosta, M.; Braithwaite, T.; Greuter, T.; et al. ECCO Guidelines on Extraintestinal Manifestations in Inflammatory Bowel Disease. J Crohns Colitis **2024**, 18, 1–37, doi:10.1093/ecco-jcc/jjad108.

Supplementary Table S2. Sex-based differences in sustained clinical remission and response concerning the type of advanced medical agent used.

Parameter	Males				Females			
	VDZ (N/Total) [1]	UST (N/Total) [1]	TOFA (N/Total) [1]	P-value [2]	VDZ (N/Total) [1]	UST (N/Total) [1]	TOFA (N/Total) [1]	P-value [3]
**Sustained clinical remission**	17.4% (24/138)	6.4% (5/78)	8.6% (5/58)	**0.024**	14.7% (15/102)	5.2% (5/96)	13.3% (10/75)	0.557
**Sustained clinical response**	58.4% (73/125)	20.3% (13/64)	19.6% (10/51)	**< 0.001**	55.9% (52/93)	24.1% (19/79)	26.6% (17/64)	**< 0.001**
**Sex-based comparison, remission (P-value [4])**	0.577	0.735	0.394	-	0.577	0.735	0.394	-
**Sex-based comparison, response (P-value [4])**	0.714	0.594	0.382	-	0.714	0.594	0.382	-

Acronyms: VDZ: vedolizumab; UST: ustekinumab; TOFA: tofacitinib; N: sample size.

Notes:
[1] In parentheses, the sample size used to calculate the data is indicated, representing the number of subjects with evaluations available at every time point to compute the differential necessary for identifying the outcome of interest.
[2] The test evaluates whether the variables of interest (sustained clinical remission and response) are differently distributed based on the type of medication administered (VDZ, UST, or TOFA), with the evaluation being carried out for males, with an alpha error threshold set at 5%. Significant values ($P < 0.05$) are boldly highlighted for easier reference.
[3] The test evaluates whether the variables of interest (sustained clinical remission and response) are differently distributed based on the type of medication administered (VDZ, UST, or TOFA), with the evaluation being carried out for females, with an alpha error threshold set at 5%. Significant values ($P < 0.05$) are boldly highlighted for easier reference.
[4] The test evaluates potential differences for every drug (i.e., VDZ, UST, and TOFA) between the subgroups considered (males and females), with an alpha error threshold set at 5%. Significant values ($P < 0.05$) are boldly highlighted for easier reference. The test employed in this setting was the χ^2 or Fisher's exact test, where appropriate.

Supplementary Table S3. Clinical and demographic differences at baseline between males and females late remitters.

**Parameter**	**Males** (N=58)	**Females** (N=38)	**P-value** [1]
**Age**	48 (35.75 – 58.25)	51.5 (42.75 – 60.25)	0.383
**Smoking status**			
Active	8 (13.8%)	2 (5.3%)	0.740
Not smoker	27 (46.6%)	18 (47.4%)	
Former smoker	23 (39.7%)	18 (47.4%)	
**Appendectomy** (*yes*)	4 (6.9%)	0 (0%)	0.098 [2]
**UC duration** (years)	11 (5 – 15.25)	7 (3 – 19.25)	0.335
**Montreal classification**			
E1 (proctitis)	2 (3.4%)	1 (2.6%)	0.663
E2 (left colitis)	24 (41.4%)	18 (47.4%)	
E3 (pancolitis)	32 (55.2%)	19 (50%)	
**Active treatment**			
Vedolizumab	38 (65.5%)	17 (55.3%)	**0.044** [2]
Ustekinumab	10 (17.2%)	8 (21.1%)	0.640 [2]
Tofacitinib	10 (17.2%)	13 (34.2%)	0.057 [2]
**Previous treatments** [2]			
Infliximab	46 (79.3%)	33 (86.8%)	0.344 [2]
Adalimumab	26 (44.8%)	12 (31.6%)	0.194 [2]
Golimumab	13 (22.4%)	2 (5.3%)	**0.024** [2]
Vedolizumab	10 (17.2%)	28 (73.7%)	0.284 [2]
Ustekinumab	2 (3.4%)	1 (2.6%)	0.822 [2]
**5-ASA** (*yes*)	57 (98.3%)	37 (97.4%)	0.761 [2]
**Steroids** (*yes*)	50 (86.2%)	35 (92.1%)	0.375 [2]
**Thiopurines** [3] (*yes*)	29 (50%)	11 (28.9%)	**0.041** [2]
**Methotrexate** [3] (*yes*)	8 (13.8%)	1 (2.6%)	0.067 [2]
**Latent TBC** (*yes*)	2 (3.4%)	1 (2.6%)	0.822 [2]
**HBV infection** (*yes*)	0 (0%)	0 (0%)	N.A.
**PMS**	5 (4 – 6.25)	5 (4 – 6.25)	0.724
**Faecal calprotectin** (*μg/g*)	666 (345 – 1235) [43] [3]	986 (451.5 – 1827.75) [22] [3]	0.212
**CRP** (*mg/dL*)	3.2 (2.1 – 12) [55] [3]	3.4 (2 – 12.75) [36] [3]	0.994
**Mayo endoscopic score**	2 (2 – 3) [57] [3]	2 (2 – 3) [36] [3]	0.285
**ESR** (*mm/h*)	16 (7 – 32) [39] [3]	31 (15 – 43) [20] [3]	0.109

Continuous variables are presented as median (interquartile range), while categorical or ordinal variables are expressed as frequencies, i.e., counts (percentage of the total in the subgroup considered, male or female).

Acronyms: N: sample size; PMS: partial Mayo score; CRP: C-reactive protein; ESR: erythrocyte sedimentation rate; N.A.: not applicable; 5-ASA: 5-aminosalicylic acid; HBV: hepatitis B virus; TBC: tuberculosis.

Notes:
[1] The test evaluates potential differences between the two subgroups considered (males and females), with an alpha error threshold set at 5%.
[2] The test employed in this setting was the χ^2 or Fisher's exact test, where appropriate.
[3] In square brackets, the sample size for these specific variables is indicated, showing the number of cases available for comparison between the two study groups. Where not explicitly stated, it is implied that the specific data were available for the total sample size of the late remitters.

Supplementary Table S4. Sex-based correlation analysis among the main clinical-demographic variables and the endoscopic disease severity at baseline and the end of the retrospective timeframe.

	**Males**		**Females**	
**Parameter (wk)** [1]	**Mayo ES (0 wk)**[2] **[N]**	**Mayo ES (48 wk)** [2] **[N]**	**Mayo ES (0 wk)**[2] **[N]**	**Mayo ES (48 wk)** [2] **[N]**
**FC (0 wk)**	**0.140** *** [220]	0.045 # [59]	**0.159** *** [214]	0.044 # [51]
**CRP (0 wk)**	**0.183** *** [277]	0.098 # [75]	**0.101** * [273]	0.078 # [68]
**PMS (0 wk)**	**0.282** *** [293]	0.062 # [81]	**0.193** *** [290]	0.166 # [70]
**ESR (0 wk)**	**0.228** *** [172]	0.125 # [54]	**0.208** *** [290]	0.108 # [45]
**FC (8 wk)**	-	**0.274 * [39]**	-	**0.394 *** [45]**
**FC (24 wk)**	-	**0.314 *** [48]**	-	**0.321 *** [43]**
**FC (48 wk)**	-	**0.412 *** [55]**	-	**0.437 *** [48]**
**CRP (8 wk)**	-	0.115 # [63]	-	**0.201 * [64]**
**CRP (24 wk)**	-	**0.248 *** [68]**	-	**0.276 *** [66]**
**CRP (48 wk)**	-	**0.272 *** [74]**	-	**0.403 *** [67]**
**PMS (8 wk)**	-	**0.366 *** [71]**	-	**0.263 *** [66]**
**PMS (24 wk)**	-	**0.392 *** [78]**	-	**0.332 *** [68]**
**PMS (48 wk)**	-	**0.567 *** [81]**	-	**0.411 *** [70]**
**ESR (8 wk)**	-	**0.243 * [45]**	-	**0.271 * [39]**
**ESR (24 wk)**	-	**0.341 *** [52]**	-	**0.327 *** [39]**
**ESR (48 wk)**	-	**0.374 *** [48]**	-	**0.375 ** [36]**
**Mayo ES (0 wk)**	-	**0.289 *** [80]**	-	0.181 # [66]

Acronyms: Wk: weeks; N: sample size; ES: endoscopic score; FC: faecal calprotectin; PMS: partial Mayo score; CRP: C-reactive protein.

Notes:

[1] The time points for calculating the variable are as follows: baseline (0 weeks), after 8 weeks, 24 weeks, and 48 weeks. Among the parameters tested (not shown because no significant results were found in any subgroup presented in the table) were additional variables, including age and disease duration.

[2] In each row, Kendall's τ correlation coefficient is displayed in sequence, followed by the statistical significance level indicated as * $P < 0.05$, ** $P < 0.01$, *** $P < 0.001$, or $^{\#}$ $P > 0.05$. Finally, the sample size used for the correlation analysis is shown in square brackets. The test presents with an alpha error threshold set at 5%. Significant values ($P < 0.05$) are boldly highlighted for easier reference. The strength of the association indicated by Kendall's τ was interpreted using the following thresholds: $0.0 < \tau < 0.1$ indicates no correlation, $0.1 \leq \tau < 0.3$ represents low correlation, $0.3 \leq \tau < 0.5$ indicates medium correlation, $0.5 \leq \tau < 0.7$ signifies high correlation, and $0.7 \leq \tau \leq 1.0$ reflects very high correlation.

Supplementary Table S5. Steroid use stratified by sex (males and females) at the four-time points where the data is available: baseline (T0), 8 weeks (T1), 24 weeks (T2), and 48 weeks (T3).

Group	Males [1]				Females [1]			
	T0	T1	T2	T3	T0	T1	T2	T3
**Overall sample**	86% (258/300)	41.3% (123/298)	24.7% (74/299)	**11.7% (34/290)** †	86.1% (260/302)	41.9% (126/301)	26.2% (78/298)	**18.7% (35/187)** †
**VDZ**	87.2% (130/149)	51% (75/147)	24.2% (36/149)	12.9% (19/147)	92.8% (103/111)	49.1% (54/110)	26.2% (28/107)	22.7% (20/88)
**UST**	93.3% (84/90)	35.6% (32/90)	22.5% (20/89)	6.9% (6/87)	95.1% (98/103)	45.6% (47/103)	29.1% (30/103)	16.1% (9/56)
**TOFA**	72.1% (44/61)	26.2% (16/61)	29.5% (18/61)	16.1% (9/56)	67% (59/88)	28.4% (25/88)	22.7% (20/88)	14% (6/43)

Acronyms: N: sample size; VDZ: vedolizumab; UST: ustekinumab; TOFA: tofacitinib.

Notes:
† The test evaluates potential differences between the two subgroups considered (males and females), with an alpha error threshold set at 5%. Significant values ($P < 0.05$) are boldly highlighted for easier reference. The test employed in this setting was the χ^2 or Fisher's exact test, where appropriate.
[1] In parentheses, the sample size used to calculate the data indicates the number of subjects with evaluations available.

Supplementary Table S6. Adverse events (AEs) recorded in the retrospective follow-up of patients included in the analysis in descending order of frequency.

AE [1]	Frequency	Percentage (%) over the total sample (N=602)
Allergy	3	0.5
Arthropaty	2	0.33
Herpes Zoster	2	0.33
Pneumonia	2	0.33
Fatigue	2	0.33
Alopecia	1	0.16
De Quervein thyroiditis	1	0.16
Erythema nodosum	1	0.16
Dermatitis	1	0.16
Clostridium difficile infection	1	0.16
Campylobacter jejuni infection	1	0.16
Renal vein thrombosis	1	0.16
Cough	1	0.16
Headache	1	0.16
Abdominl pain	1	0.16

[1] AEs were classified and assessed in accordance with the World Health Organization (WHO) classification (J Public Health Res 2013;2:e29 [1]).

Supplementary reference

1. Larizgoitia, I.; Bouesseau, M.-C.; Kelley, E. WHO Efforts to Promote Reporting of Adverse Events and Global Learning. J Public Health Res **2013**, 2, e29, doi:10.4081/jphr.2013.e29.

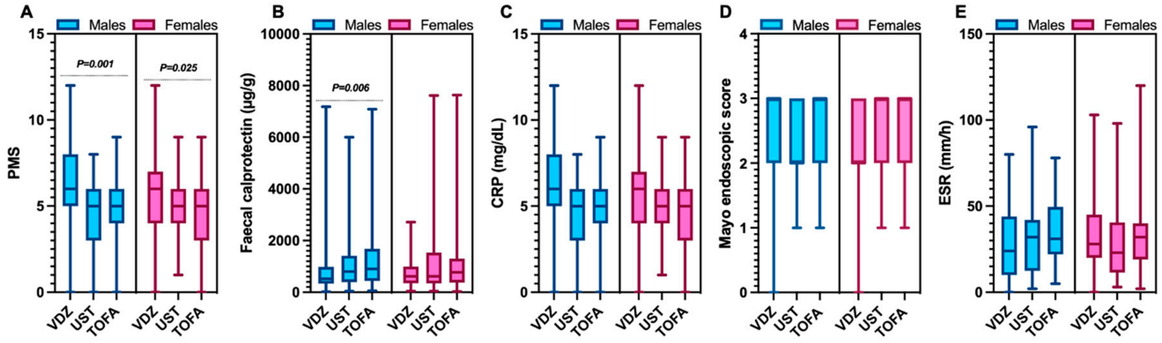

Supplementary Figure S1. Levels of partial Mayo score (PMS, **A**), faecal calprotectin (**B**), C-reactive protein (CRP, **C**), Mayo endoscopic score (**D**), and erythrocyte sedimentation rate (ESR, **E**) at baseline (T0) in the overall sample, stratified by sex (males and females). The variables are presented as median (interquartile range). The test evaluates potential differences between the two subgroups considered (males and females), with an alpha error threshold set at 5%. Significant values ($P < 0.05$) are boldly highlighted for easier reference. The sample size of each subgroup can be deduced from Table 2.
